# The Predictive Level of Body Image and Self-Esteem in Emerging Adulthood on Eating Attitudes: The Mediating Role of Life Satisfaction

**DOI:** 10.3390/healthcare14091164

**Published:** 2026-04-27

**Authors:** Özge Sarıca Acaröz, Mehmet Çakıcı

**Affiliations:** 1 Doctoral Program in Clinical Psychology, Cyprus Health and Social Sciences University, Morphou 99750, Türkiye; 2 Faculty of Social Sciences and Humanities, Cyprus Health and Social Sciences University, Morphou 99750, Türkiye; mehmet.cakici@kstu.edu.tr

**Keywords:** eating attitude, body image, self-esteem, life satisfaction, emerging adulthood

## Abstract

**Background/Objectives**: Psychological variables such as body image, self-esteem, and life satisfaction have become important research topics in recent years, particularly in their relationships with individuals’ eating attitudes. The purpose of this study is to examine the predictive effect of body image and self-esteem on eating attitudes in emerging adults and to evaluate the mediating role of life satisfaction in this relationship. **Method**: The study included 402 participants aged 18–30. Data were collected using the Eating Attitudes Test (EAT-26), the Rosenberg Self-Esteem Scale (RSS), the Body Image Scale (BIS), and the Life Satisfaction Scale (LSS). The analyses included correlational analyses to examine relationships among eating attitudes, body image, self-esteem, and life satisfaction; multivariate regression to test the predictive roles of body image, self-esteem, and life satisfaction on eating attitudes; and structural equation modeling to evaluate the mediating role of life satisfaction in the relationships between body image, self-esteem, and eating attitudes. **Result**: The correlational analysis revealed that eating attitudes are associated with body image, life satisfaction, and self-esteem. Body image was identified as the most influential predictor of eating attitudes. Structural equation modeling indicated that life satisfaction mediates the relationship between self-esteem and eating attitudes. **Conclusions**: Body image, self-esteem, and life satisfaction play a crucial role in shaping eating attitudes among emerging adults. The mediating effect of life satisfaction underscores psychological well-being as a key regulatory factor rather than solely an outcome. Promoting positive body image, strengthening self-esteem, and enhancing life satisfaction through preventive and psychoeducational programs may help protect against disordered eating attitudes in this population.

## 1. Introduction

Emerging adulthood is a critical developmental stage spanning approximately 18–30 years, during which individuals become more aware of their physical appearance, social comparisons intensify, and self-perception is shaped [1,2,3]. During this period, psychosocial variables such as body image, self-esteem, and life satisfaction play an important role in the development of healthy or unhealthy eating attitudes [4,5]. Recently, with the increasing influence of media and social media, the pressures on young people’s body image and self-perception can lead to disturbances in their eating attitudes [6]. Eating attitudes extend beyond mere physiological need; they are shaped by cultural, psychological, and social factors, which contribute to the development of unhealthy habits such as unconscious, unbalanced, and rapid eating [7,8]. This situation disrupts individuals’ attitudes toward eating, encompassing their thoughts, feelings, and behaviors related to food [9]. These disturbances are more pronounced in individuals with eating disorders, and unrealistic beliefs can play a role in this process.

Eating attitude refers to the tendency that guides an individual’s thoughts, feelings, and behaviors related to food [10]. Previous studies have demonstrated that eating attitudes are associated with a wide range of psychological and behavioral variables. For instance, excessive adherence to a healthy lifestyle, often conceptualized as orthorexia tendencies, has been linked to maladaptive eating patterns [11]. More recently, cognitive factors, such as rumination, have gained attention for their role in perpetuating maladaptive thought patterns related to food and body image [12]. Moreover, self-esteem and body image have consistently been identified as strong predictors of eating attitudes, with lower self-worth and negative body evaluations increasing vulnerability to unhealthy eating attitudes [13,14,15]. Similarly, social media use has been shown to influence eating attitudes through mechanisms such as body comparison and internalization of unrealistic appearance ideals [16]. Traumatic life experiences also appear to contribute to the development of the eating attitude by disrupting emotion regulation processes [17].

Another important factor influencing eating attitudes is body image. Individuals’ evaluations of their bodies and their genetic characteristics play a role in shaping healthy or unhealthy eating attitudes [14,18]. Negative body image arises when an individual perceives a difference between their current body appearance and their ideal body image, and this dissatisfaction can lead to compensatory behaviors such as unhealthy diets and excessive exercise [19,20]. These behaviors are often associated with weight concerns, low self-esteem, and eating disorders. It is observed that as body perception increases, the negative aspects of eating attitudes decrease [21].

Rosenberg defined self-esteem in 1965 as an individual’s positive or negative self-evaluation. Low self-esteem is considered a risk factor for eating disorders and disordered eating behaviors [22,23]. It is stated that low self-esteem negatively affects eating attitudes [14,24]. Recent research has further highlighted the critical role of low self-esteem in the development of eating disorders. Low self-esteem is an important psychological factor that negatively affects eating attitudes, and it appears to be linked to life satisfaction and eating attitudes at multiple levels [24].

Disordered eating attitudes can negatively impact not only an individual’s self-esteem but also their overall life satisfaction. Life satisfaction is defined as the general level of contentment an individual experiences when comparing what they have with what they aspire to achieve [25]. It represents a cognitive evaluation of one’s quality of life and reflects the extent to which individuals perceive their goals, needs, and expectations as being fulfilled [26,27]. Disordered eating attitudes appear to be consistently associated with lower levels of life satisfaction [24,28,29]. For instance, individuals who exhibit irregular eating patterns exhibit notably lower life satisfaction than those with healthier eating habits [28].

Body image, self-esteem, life satisfaction, and eating attitudes are important variables that interact and influence an individual’s physical and psychological well-being [21,22,23,24,25,26,27,28,29,30,31]. Understanding the effects of fundamental psychological constructs, such as body image and self-esteem, on eating attitudes is important for preventing risky eating attitudes. However, one of the most original aspects of this study is that it tests the mediating role of life satisfaction in these relationships. Therefore, elucidating how life satisfaction acts as a bridge between these variables will contribute significantly to both theoretical and applied psychology. Emerging adulthood is not only a transitional period but also a developmentally distinct phase characterized by identity exploration, instability, and heightened sensitivity to external evaluation, particularly regarding physical appearance and social approval [32,33]. Compared to adolescence, individuals in this period experience increased autonomy and responsibility, while still actively constructing their identity, which makes self-evaluative processes such as body image and self-esteem more salient and cognitively elaborated [34,35]. In contrast to later adulthood, where identity structures and self-concept tend to stabilize, emerging adults are more vulnerable to fluctuations in life satisfaction and self-worth due to ongoing life transitions [36,37]. Within this context, life satisfaction may function as a more dynamic and influential psychological mechanism that links body image and self-esteem behavioral outcomes such as eating attitudes [38,39]. While similar relationships may exist in other age groups, the mediating role of life satisfaction is expected to be more pronounced in emerging adulthood due to the developmental prominence of identity formation and subjective well-being during this period [36]. The aim of this study is to examine the predictive role of body image and self-esteem in emerging adulthood on eating attitudes and to evaluate the possible mediating effect of life satisfaction in this relationship. The study may serve as a guide in the design of preventive mental health programs aimed at helping emerging adults develop a healthy body image and eating attitudes. It is also important for developing multivariate models that support the individual’s holistic psychological well-being.

## 2. Materials and Methods

### 2.1. Research Model

In this study, the correlational survey model, a quantitative research method, was employed to examine the relationships among eating attitudes, body image, self-esteem, and life satisfaction. The correlational survey model is a research approach used to assess relationships among two or more variables. This model focuses on analyzing the strength of relationships among variables and how these variables influence one another [40].

### 2.2. Population and Sample

The study population consisted of volunteers aged 18–30 years (corresponding to birth years between 1995 and 2007) residing in North Cyprus. Participants were selected via quota sampling from a purposive sample, with attention to demographic characteristics best suited to the study’s purpose [40]. The inclusion criteria for the study were: (a) being between the ages of 18 and 30, (b) residing in North Cyprus, and (c) voluntarily agreeing to participate in the study. The exclusion criteria included: (a) providing incomplete or inconsistent responses, and (b) being outside the specified age range. Data were collected from individuals in various community settings, including universities, social environments, and online platforms, to increase sample diversity. The sample size was calculated using the formula for unknown populations [40]. Assuming a 95% confidence level (Z = 1.96), a 5% margin of error (d = 0.05), and a conservative proportion estimate (*p* = 0.5), the minimum required sample size was determined to be 384 participants. A total of 402 individuals participated in the study, exceeding the minimum requirement and thereby enhancing the statistical power and reliability of the findings. In addition, socioeconomic status variables (e.g., income level and parental education) were not collected in the present study. The primary reason for this is that such information is often considered sensitive, which may lead participants to refrain from responding, thereby increasing the risk of lower response rates and missing data. Therefore, these variables were not included in order to enhance participant engagement and preserve data completeness. Information regarding psychiatric medication use was obtained through a self-report demographic form. However, no exclusion criteria were applied based on psychological or psychiatric conditions, as the study aimed to reflect a naturalistic, non-clinical sample of emerging adults.

### 2.3. Data Collection Tools

#### 2.3.1. Socio-Demographic Information Form

The demographic information form, developed by the researcher, comprises questions designed to collect participants’ demographic data, including age, gender, education level, body height, body weight, use of psychiatric medication, and history of psychiatric diagnosis.

#### 2.3.2. Eating Attitude Test-26 (EAT-26)

This test was developed to enable individuals to assess their eating attidues attitudes. It was developed by Garner and Garfinkel (1979) [41] and later validated and reliability tested in Turkish by Ergüney-Okumuş and Sertel-Berk (2020) [42]. The scale has three subscales: preoccupation with eating, restriction, and social pressure. The EAT-26 consists of 26 items and is scored on a scale of 0–53. The cutoff point is set at 20 points, indicating that individuals scoring 20 or higher have impaired eating attitudes. The Cronbach’s alpha coefficient for the EAT-26 was 0.84 [42]. It was also 0.83 in this study.

#### 2.3.3. Rosenberg Self-Esteem Scale (RSS)

The scale was developed by Rosenberg (1965) [43], and the Turkish adaptation was first done by Çuhadaroğlu (1986) [44] and then by Korkmaz (2022) [45]. It comprises 5 positive and 5 negative items, for a total of 10 items. The total score for general self-esteem is calculated from 10 items; however, negative items must be converted to positive items for scoring. The scale is scored on a 4-point Likert scale. A high score on the scale indicates high self-esteem. The positive items are 1, 3, 4, 7, and 10; the negative items are 2, 5, 6, 8, and 9. The test-retest reliability of the scale was 0.71, and Cronbach’s Alpha was 0.84 [45]. For this study, the coefficient was 0.78.

#### 2.3.4. Body Image Scale (BIS)

Developed by Secord and Jourard (1953) [46]. It measures the level of satisfaction individuals have with their body parts and functions. The Turkish validity and reliability study was conducted by Hovardaoğlu (1993) [47]. It consists of 40 items on a 5-point Likert scale. The scale is scored on a 1–5 scale. A high score on the scale indicates greater dissatisfaction with body parts and functions. The Cronbach’s alpha coefficient for the scale was 0.91 [47]. The Cronbach’s alpha value for this study was 0.95.

#### 2.3.5. Life Satisfaction Scale (LSS)

The scale was developed by Diener, Emmons, Larsen, and Griffin (1985) to assess individuals’ overall life satisfaction [48]. The Turkish adaptation was conducted by Dağlı and Baysal (2016) [49]. The scale focuses on the cognitive component of subjective well-being and measures the extent to which individuals find their lives generally satisfying. The scale consists of 5 items and a 7-point Likert scale. A high score indicates a high level of life satisfaction. The internal consistency coefficient of the Turkish form was reported as 0.88 [49]. For this study, the coefficient was 0.86.

### 2.4. Data Collection Process

In the study, both online and face-to-face surveys were administered to individuals aged 18–30. Face-to-face data collection was conducted in community-based settings, such as university campuses and social environments, in order to reach individuals who may have limited access to or lower participation rates in online surveys. The online survey, on the other hand, was used to facilitate broader and more flexible participation. The combined use of these methods was intended to increase response rates, enhance sample diversity, and reduce potential sampling bias. The survey comprised valid and reliable scales assessing body image, self-esteem, life satisfaction, and eating attitudes. Body Mass Index (BMI) was calculated using the standard formula by dividing participants’ self-reported body weight (kg) by the square of their height (m^2^). In addition, variables such as maximum body weight, lowest body weight in adulthood, and ideal body weight were obtained based on participants’ self-reports and were not calculated using standardized formulas. Data were collected on a voluntary basis, and all responses were evaluated anonymously. To ensure methodological consistency, all participants were presented with the same set of instruments in the same order and with identical instructions, regardless of whether the data were collected online or face-to-face. This standardization aimed to minimize potential variability arising from different modes of data collection. In addition, although data were collected through multiple methods and from diverse settings, efforts were made to maintain uniform administration procedures across all contexts. This approach was adopted to enhance the comparability and reliability of the collected data.

### 2.5. Statistical Analysis

Statistical analyses were conducted using SPSS 27.0. The normality of the variables was evaluated using both Kolmogorov–Smirnov test results and skewness–kurtosis values. Although the Kolmogorov–Smirnov test yielded statistically significant results (*p* < 0.05), this test is known to be highly sensitive to large sample sizes. Therefore, skewness and kurtosis values were also considered. All variables were within the acceptable range of ±3, indicating approximate normality [50]. Accordingly, parametric tests were used in the analyses. Cronbach’s alpha coefficients were calculated to assess the internal consistency of the scales. The alpha coefficients were 0.838 for EAT-26, 0.786 for RSS, 0.954 for BIS, and 0.868 for LSS. Pearson correlation analysis was conducted to examine the relationships among eating attitudes, body image, self-esteem, and life satisfaction. Subsequently, multiple linear regression analysis was performed to assess the predictive roles of body image, self-esteem, and life satisfaction on eating attitudes. Descriptive statistics, including means ($\bar{X}$) and standard deviations (S), were calculated for all study variables. To further examine the hypothesized relationships, structural equation modeling (SEM) was conducted to test the mediating role of life satisfaction in the relationship between body image and self-esteem and eating attitudes. The model fit was evaluated using standard fit indices, including χ^2^/df, CFI, TLI, and RMSEA.

### 2.6. Ethical Compliance

This study was approved by the Scientific Ethics Committee on 17 September 2024 (file number KSTU//2024/348). Following approval, all participants intending to participate were informed about the study, and their informed consent was obtained prior to data collection, thereby demonstrating their voluntary participation.

## 3. Results

A total of 402 individuals participated in this study. Among participants, 78.60% were female, and 21.40% were male. Regarding age distribution, 79.10% of participants were aged 18–24, and 20.90% were aged 25–30. Regarding educational level, 10.00% were high school graduates, while 90.00% were university graduates. Regarding weight satisfaction, 45.00% of participants reported being satisfied with their current weight, whereas 55.00% reported dissatisfaction. In terms of psychiatric medication use, 17.40% indicated that they were currently using psychiatric medication, while 82.60% were not. Finally, regarding the history of psychiatric diagnosis, 8.20% reported having received a psychiatric diagnosis, whereas 91.80% reported no such diagnosis.

Table 1 presents the descriptive statistics of the study variables for the overall sample. Means and standard deviations are reported.

Table 2 presents correlations between participants’ EAT-26, RSS, BIS, and LSS scores and anthropometric measurements. According to the correlation analysis results shown in Table 3, there was a significant correlation between Eating Preoccupation and body weight (r = 0.188, *p* < 0.000), BMI (r = 0.174, *p* < 0.000), highest body weight (r = 0.225, *p* < 0.000), and lowest body weight in adulthood (r = 0.122, *p* < 0.014). Social pressure was negatively correlated with body weight (r = −0.291, *p* < 0.000), BMI (r = −0.303, *p* < 0.000), highest body weight (r = −0.243, *p* < 0.000), lowest body weight in adulthood (r = −0.285, *p* < 0.000), and ideal body weight (r = −0.225, *p* < 0.000). Negative correlations were found between RSS and body weight (r = −0.101, *p* < 0.044) and highest body weight (r = −0.109, *p* < 0.028), while no significant relationship was observed with other anthropometric measurements. A positive correlation was observed between EAT-26 and maximum body weight (r = 0.126, *p* < 0.012), whereas no significant associations were observed with other anthropometric measurements. A significant negative correlation was observed between BIS and the lowest body weight in adulthood (r = −0.103, *p* < 0.039), whereas no significant associations were observed with other anthropometric measurements.

Table 3 presents correlations among participants’ EAT-26, RSS, BIS, and LSS scores. In Table 3, a significant negative relationship was observed between EAT-26 and BIS (r = −0.221, *p* = 0.000). Similarly, a significant negative relationship was found between Eating Preoccupation and BIS (r = −0.278, *p* < 0.000). Negative correlations were found between RSS and Eating Preoccupation (r = −0.157, *p* < 0.002) and EAT-26 (r = −0.111, *p* < 0.026), no significant relationship was found between RSS and Restriction (r = 0.002, *p* > 0.972) and Social Pressure (r = −0.030, *p* > 0.552). Negative significant correlations were found between BIS and Eating Preoccupation (r = −0.278, *p* < 0.000) and EAT-26 (r = −0.221, *p* < 0.000), but no significant relationship was observed with Restriction (r = −0.073, *p* > 0.143) and Social Pressure (r = −0.030, *p* > 0.555). Significant relationships were found between LSS and Eating Preoccupation (r = −0.182, *p* < 0.000), EAT-26 (r = −0.145, *p* < 0.004), and BIS (r = 0.517, *p* < 0.000).

Table 4 presents the predictive power of participants’ scores on the Rosenberg Self-Esteem Scale, Body Image Scale, and Life Satisfaction Scale for their Eating Attitude Test scores. According to the regression analysis in Table 4, participants’ Rosenberg Self-Esteem (β = −0.041, *p* < 0.436) and Life Satisfaction Scale (β = −0.035, *p* < 0.542) scores do not predict Eating Attitude Test scores in a statistically significant manner. Participants’ Body Image Scale scores significantly predict Eating Attitude Test scores (β = −0.189, *p* < 0.001). The model explaining the variance in participants’ scores on the Rosenberg Self-Esteem Scale, Body Image Scale, and Life Satisfaction Scale, which predicted Eating Attitude Test scores, accounted for 4.44%.

**Figure 1:**
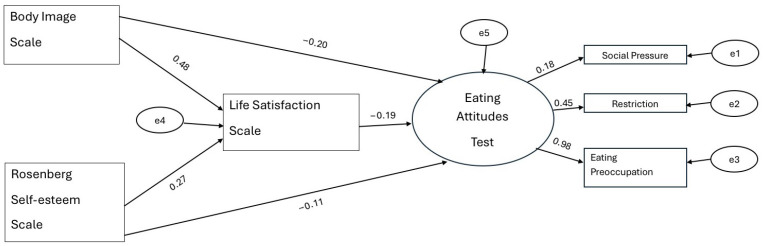
Structural equation model showing the relationships among body image, self-esteem, life satisfaction, and eating attitudes. Model fit indices indicated an acceptable fit (χ^2^/df = 2.94, RMSEA = 0.068, CFI = 0.95, TLI = 0.93, SRMR = 0.048). Note: χ^2^/df = 2.94, RMSEA = 0.068, CFI = 0.95, TLI = 0.93, SRMR = 0.048.

Figure 1 presents the structural equation model examining the relationships among participants’ Rosenberg Self-Esteem Scale, Body Image Scale, Life Satisfaction Scale, and Eating Attitude Test scores. As shown in Figure 1, Body Image Scale scores significantly predicted Life Satisfaction Scale scores in a positive direction (β = 0.48, *p* < 0.05). Similarly, Rosenberg Self-Esteem Scale scores were found to significantly predict Life Satisfaction Scale scores in a positive direction (β = 0.27, *p* < 0.05). In turn, Life Satisfaction Scale scores significantly predicted Eating Attitude Test scores in a negative direction (β = −0.19, *p* < 0.05). Regarding direct effects, Body Image Scale scores significantly predicted Eating Attitude Test scores in a negative direction (β = −0.20, *p* < 0.05), whereas Rosenberg Self-Esteem Scale scores did not significantly predict Eating Attitude Test scores (β = −0.11, *p* > 0.05). Based on these findings, Life Satisfaction Scale scores were found to play a mediating role in the relationship between Body Image and Eating Attitudes, while life satisfaction may function as a mediating variable in the relationship between self-esteem and eating attitudes. Additionally, the latent construct of Eating Attitudes Test was significantly represented by its sub-dimensions, including social pressure (β = 0.18), restriction (β = 0.45), and eating preoccupation (β = 0.98), indicating an adequate measurement structure.

## 4. Discussion

In the study, body image emerged as one of the strongest determinants of eating attitudes and contributed to unhealthy eating behaviors. Previous studies have reported that media exposure, sociocultural pressures, and the internalization of idealized body standards are associated with unhealthy eating attitudes through increased body dissatisfaction [51,52,53]. In particular, exposure to idealized body representations has been shown to exacerbate body dissatisfaction among young individuals, which in turn leads to disturbances in eating attitudes [54,55,56]. Increased body weight has likewise been associated with declines in both body image and self-esteem [57,58]. Although body dissatisfaction and more unhealthy eating attitudes are commonly observed among individuals with obesity [59,60], some findings suggest that the strength and direction of this relationship may vary depending on cultural context, sample characteristics, and measurement instruments [61,62,63]. Negative cognitive preoccupation with the body has been proposed as a core psychological mechanism underlying maladaptive eating attitudes [64,65]. In addition, the significant association identified between eating attitudes and anthropometric characteristics suggests that variables such as body weight and body composition may have acted as potential confounding factors, influencing not only eating attitudes but also related psychological constructs, including body image, self-esteem, and life satisfaction.

Another key finding of the present study indicates a relationship between self-esteem and eating attitudes. Individuals with higher self-esteem were more likely to exhibit healthy eating attitudes, whereas unhealthy eating attitudes were reported more frequently among individuals with lower self-esteem [13,66,67]. However, some studies have failed to identify a significant association between self-esteem and eating attitudes [22,68]. These inconsistencies may be attributable to differences in age, sex, cultural context, and the measurement instruments employed. Overall, the literature suggests that self-esteem does not operate as an isolated determinant of eating attitudes but rather exerts its influence alongside multiple factors, including social support, body image, and life satisfaction [13,23,69,70,71]. It is well established that underlying psychological conditions, such as depression and anxiety, can influence both self-esteem and eating behaviors [72,73]. In the present study, a proportion of participants reported using psychiatric medications, which may indicate the presence of such conditions. These factors may have systematically affected the psychological variables assessed, thereby potentially influencing the observed relationships.

The present study identified life satisfaction as a significant mediator in the relationship between self-esteem and eating attitudes. Specifically, higher levels of self-esteem were associated with increased life satisfaction, which in turn contributed to healthier eating attitudes. This finding suggests that life satisfaction may serve as an important psychological mechanism through which self-related evaluations influence eating-related behaviors. Consistent with previous research, higher life satisfaction has been linked to more adaptive eating patterns, improved psychological well-being, and more positive self-perceptions [72,73]. In addition, life satisfaction has been found to be positively associated with body image, indicating that individuals with greater life satisfaction tend to report more favorable body evaluations and fewer dysfunctional eating attitudes [71,74]. It has also been suggested that life satisfaction may reduce negative self-evaluations, alleviate psychological distress, and act as a protective factor against maladaptive eating behaviors [70,75]. Particularly during emerging adulthood, life satisfaction may play a buffering role in the relationship between body image and eating attitudes by mitigating the effects of social comparison processes. Given the increasing influence of sociocultural pressures and idealized body representations in contemporary media, life satisfaction appears to function as a key protective factor that reduces vulnerability to these external influences [53]. Furthermore, physical activity is known to be significantly associated with both psychological well-being and eating behaviors [76]. The omission of this variable represents an important limitation, as physical activity may have confounded the relationships among body image, self-esteem, life satisfaction, and eating attitudes.

In the present study, the descriptive findings indicated moderate levels of body image, self-esteem, life satisfaction, and eating attitudes across the overall sample. This suggests that concerns related to body image and eating behaviors are not uncommon in emerging adulthood. Such patterns may be associated with the widespread influence of sociocultural pressures and the increasing prevalence of appearance-focused content on media and social networking platforms [77,78]. Therefore, sociocultural influences and media-related pressures should be given greater consideration when examining eating attitudes and body image. Furthermore, in emerging adulthood, body image should be conceptualized not as a fixed trait but as a developmental and contextual process, with individuals’ body evaluations evolving over time and gradually shifting from aesthetic concerns toward functionality, health, and the quality of the relationship with the body [1,79,80].

This study also has several strengths. First, examining body image, self-esteem, life satisfaction, and eating attitudes within the same model provides a more comprehensive understanding of how these variables interact. The relatively large sample size and the use of valid and reliable measurement tools strengthen the scientific rigor of the findings. Moreover, the application of structural equation modeling offers an original contribution to the literature by demonstrating the mediating role of life satisfaction.

However, several limitations should be considered when interpreting the findings. First, the cross-sectional design precludes causal inferences regarding the relationships among variables. More importantly, several potential confounding factors were not controlled for in the analyses. Anthropometric characteristics, which were found to be significantly associated with eating attitudes, may have influenced not only eating behaviors but also related psychological variables such as body image, self-esteem, and life satisfaction. Similarly, physical activity levels—known to be closely associated with both psychological well-being and eating behaviors—were not assessed in the present study. Therefore, the absence of physical activity data may have influenced the observed relationships among variables, and the findings should be interpreted with caution in this regard. In addition, the use of multiple data collection methods (online and face-to-face) may have introduced methodological variability, despite efforts to standardize administration procedures. Furthermore, the absence of exclusion criteria based on psychological or psychiatric conditions may have increased variability in responses. In addition, a notable proportion of participants reported using psychiatric medications, which may reflect underlying mental health conditions such as depression or anxiety. These factors may have systematically influenced all study variables and, therefore, may have affected the observed relationships. Furthermore, the sample was drawn from a single cultural context and exhibited an unequal gender distribution, which may limit the generalizability of the findings. The use of self-report measures also raises the possibility of response and social desirability biases. Accordingly, the findings should be interpreted with caution. Future research is recommended to employ longitudinal designs, control for relevant variables such as physical activity, anthropometric characteristics, and mental health status, and include more diverse and balanced samples to enhance the robustness and generalizability of the results.

## 5. Conclusions

This study highlights the significant role of psychological variables—specifically body image, self-esteem, and life satisfaction—in shaping eating attitudes among emerging adults. The findings demonstrate that eating attitudes are influenced not only by biological and environmental factors but also by psychosocial dynamics, underscoring the multifaceted nature of risk for disordered eating. In particular, the mediating role of life satisfaction underscores that psychological well-being should be considered not merely as an outcome but also as a regulatory mechanism that affects both behavior and emotional processes.

The results suggest that preventive intervention programs that promote positive body image, strengthen self-esteem, and enhance life satisfaction may serve as protective factors against unhealthy eating attitudes. Media literacy, self-worth training, and psychoeducational initiatives that foster self-awareness could be particularly effective in this regard. Overall, this research contributes to understanding how psychological and social factors interact with eating attitudes, offering both a theoretical framework for future studies and practical insights for comprehensive mental health interventions targeting emerging adulthood. Future studies may benefit from employing longitudinal designs to better capture the causal relationships among body image, self-esteem, life satisfaction, and eating attitudes throughout emerging adulthood. Additionally, incorporating qualitative or mixed-method approaches may provide deeper insight into the cognitive and emotional processes that contribute to disordered eating risk. Expanding the sample to include different cultural contexts and clinical populations could enhance the generalizability of the findings. Furthermore, exploring additional mediators—such as social comparison, self-compassion, emotion regulation, and media literacy—may help develop more comprehensive models that explain how eating attitudes are shaped.

## Figures and Tables

**Table 1:** Descriptive Statistics for Study Variables (N = 402).

	$\bar{X}$	s
Eating Preoccupation	4.64	5.74
Restriction	2.67	3.47
Social pressure	1.66	2.37
Eating Attitudes Test (EAT-26)	8.98	8.70
Rosenberg Self-Esteem Scale (RSS)	15.93	2.15
Body Image Scale (BIS)	147.87	28.00
Life Satisfaction Scale (LSS)	16.24	4.54

Note: $\bar{X}$ = mean; s = standard deviation.

**Table 2:** Correlations between Participants’ Eating Attitude Test, Rosenberg Self-Esteem Scale, Body Perception Scale, and Life Satisfaction Scale Scores and Anthropometric Measurements (N = 402).

		Body Weight (kg)	Height (cm)	BMI (kg/m^2^)	Maximum Body Weight (kg)	Lowest Body Weight in Adulthood (kg)	Ideal Body Weight (kg)
Eating	r	0.188	0.046	0.174	0.225	0.122	0.075
Preoccupation	*p*	0.000 *	0.362	0.000 *	0.000 *	0.014 *	0.136
Restriction	r	0.024	−0.042	0.050	0.111	0.023	0.024
	*p*	0.628	0.404	0.314	0.026	0.649	0.635
Social pressure	r	−0.291	−0.096	−0.303	−0.243	−0.285	−0.225
	*p*	0.000 *	0.054	0.000 *	0.000 *	0.000 *	0.000 *
Eating Attitudes Test (EAT-26)	r	0.054	−0.013	0.052	0.126	0.012	−0.003
	*p*	0.277	0.798	0.296	0.012 *	0.812	0.954
Rosenberg Self-Esteem Scale (RSS)	r	−0.101	−0.048	−0.085	−0.109	−0.056	−0.084
	*p*	0.044 *	0.335	0.089	0.028 *	0.264	0.092
Body Image Scale (BIS)	r	−0.060	0.087	−0.103	−0.083	0.007	0.050
	*p*	0.232	0.081	0.039	0.098	0.881	0.318
Life Satisfac-tion Scale (LSS)	r	0.029	0.047	0.024	−0.007	0.031	0.086
	*p*	0.559	0.349	0.633	0.887	0.539	0.084

Note: * *p* < 0.05.

**Table 3:** Correlations between Participants’ Eating Attitude Test, Rosenberg Self-Esteem Scale, Body Image Scale, and Life Satisfaction Scale Scores (N = 402).

		Eating Preoccupation	Restriction	Social Pressure	Eating Attitudes Test (EAT-26)	Rosenberg Self-Esteem Scale (RSS)	Body Image Scale (BIS)	Life Satisfaction Scale (LSS)
Eating Preoccupation	r	1						
	*p*							
Restriction	r	0.438	1					
	*p*	0.000 *						
Social pressure	r	0.168	0.184	1				
	*p*	0.001	0.000 *					
Eating Attitudes Test (EAT-26)	r	0.880	0.738	0.457	1			
	*p*	0.000 *	0.000 *	0.000 *				
Rosenberg Self-Esteem Scale (RSS)	r	−0.157	0.002	−0.030	−0.111	1		
	*p*	0.002	0.972	0.552	0.026			
Body Image Scale (BIS)	r	−0.278	−0.073	−0.030	−0.221	0.317	1	
	*p*	0.000 *	0.143	0.555	0.000 *	0.000 *		
Life Satisaction Scale (LSS)	r	−0.182	−0.033	−0.042	−0.145	0.288	0.517	1
	*p*	0.000 *	0.514	0.403	0.004	0.000 *	0.000 *	

Note: * *p* < 0.05.

**Table 4:** The Predictive Power of Participants’ Rosenberg Self-Esteem Scale, Body Image Scale, and Life Satisfaction Scale Scores on Eating Attitude Test Scores.

	Not Std.		Std.	t	*p*	F	R^2^
	β	S.H.	Beta			*p*	AdjR^2^
(Fixed)	21.402	3.436		6.229	0.000		
Rosenberg Self-Esteem Scale (RSS)	−0.164	0.211	−0.041	−0.780	0.436	7.186	0.051
Body Image Scale (BIS)	−0.059	0.018	−0.189	−3.251	0.001 *	0.000 *	0.044
Life Satisfaction Scale (LSS)	−0.067	0.110	−0.035	−0.610	0.542		

Note: R^2^ = Coefficient of determination; AdjR^2^ = Adjusted Coefficient of Determination; F = F-statistic test. * *p* < 0.05.

## Data Availability

The data supporting the findings of this study are available upon reasonable request. Researchers interested in accessing the data can contact Özge Sarıca Acaröz at ozgesarica@hotmail.com. We are committed to promoting transparency and facilitating data sharing to further scientific investigation.

## Abbreviations

EAT-26	Eating Attitude Test-26
RSS	Rosenberg Self-Esteem Scale
BIS	Body Image Scale
LSS	Life Satisfaction Scale
